# Identification of a potentially functional circRNA–miRNA–mRNA regulatory network for investigating pathogenesis and providing possible biomarkers of bladder cancer

**DOI:** 10.1186/s12935-020-1108-3

**Published:** 2020-01-29

**Authors:** Hong-cheng Lu, Jia-qi Yao, Xiao Yang, Jie Han, Jing-zi Wang, Kun Xu, Rui Zhou, Hao Yu, Qiang Lv, Min Gu

**Affiliations:** 1 Department of Urology, The First Affiliated Hospital of Nanjing Medical University, 300, Guangzhou Road, Nanjing, 210029 Jiangsu China; 2 Department of Oncology, The First Affiliated Hospital of Nanjing Medical University, 300, Guangzhou Road, Nanjing, 210029 Jiangsu China

**Keywords:** Bladder cancer, circRNA, ceRNA, Biomarker

## Abstract

**Background:**

Circular RNAs (circRNAs) have received considerable attention in human cancer research. However, many circRNAs remain to be detected. In our study, we determined novel circRNAs and investigated their effects on bladder cancer (BCa).

**Methods:**

Microarray dataset GSE92675 was downloaded from Gene Expression Omnibus (GEO). Then, we combined computational biology with quantitative real-time polymerase chain reaction (qRT-PCR) to select related circRNAs in BCa. The selected circRNA–microRNA (miRNA)–messenger RNA (mRNA) regulatory subnetwork was determined by Gene Oncology (GO) and Kyoto Encyclopedia of Genes and Genomes (KEGG) analyses.

**Results:**

The regulatory network constructed from the microarray dataset (GSE92675) contained 49 differentially expressed circRNAs (DECs). GO and KEGG analyses showed that the MAPK and PI3K–AKT signaling pathways were statistically significant. On the basis of qRT-PCR and the degree value calculated by the cytoHubba plugin of Cytoscape, hsa_circ_0011385 was finally confirmed. The subnetwork around hsa_circ_0011385 was constructed. In addition, we created a protein–protein interaction (PPI) network composed of 67 nodes and 274 edges after removing independent nodes. GO and KEGG analyses showed that hubgenes were involved in cell cycle activities. Moreover, they could be regulated by miRNAs and play an eventful role in BCa pathogenesis.

**Conclusions:**

We proposed a novel circRNA–miRNA–mRNA network related to BCa pathogenesis. This network might be a new molecular biomarker and could be used to develop potential treatment strategies for BCa.

## Background

Bladder cancer (BCa) has become the most commonly occurring cancer of the urinary system with high mortality rates worldwide [1]. It is categorized into two types: non-muscle-invasive bladder cancer (NMIBC) and muscle-invasive bladder cancer (MIBC). Although several treatment approaches, such as surgical chemotherapy and radiation therapy, have been developed, the 5-year survival rate of patients with MIBC is only approximately 60% [2, 3]. Poor understanding of the mechanisms underlying BCa pathogenesis is one of the main reasons for this phenomenon. Mounting evidence show that circular RNAs (circRNAs) play an important role in BCa pathogenesis.

CircRNAs, which widely exist in eukaryotic cells with a covalently closed loop structure, were observed for the first time in 1976 [4]. However, they were misconceived as by-products of splicing errors due to the limitation of traditional RNA detection methods [5]. In recent years, numerous circRNAs have been detected in various cell lines and species with the application of high-throughput sequencing technology [6–8]. CircRNAs are involved in the initiation and progression of cancers. One of their molecular mechanisms is that they act as competing endogenous RNAs (ceRNAs), which serve as microRNA (miRNA) sponges. CDR1as, a classic circRNA in the ceRNA family, performs biological or pathological functions in many cancers by absorbing miR-7 as miRNA sponge with more than 70 miRNA response elements (MREs) [9–11]. CircRNAs have also been reported to act as ceRNAs that regulate gene expression in BCa [12]. However, many unknown circRNAs still remain to be investigated.

**Fig. 1:**
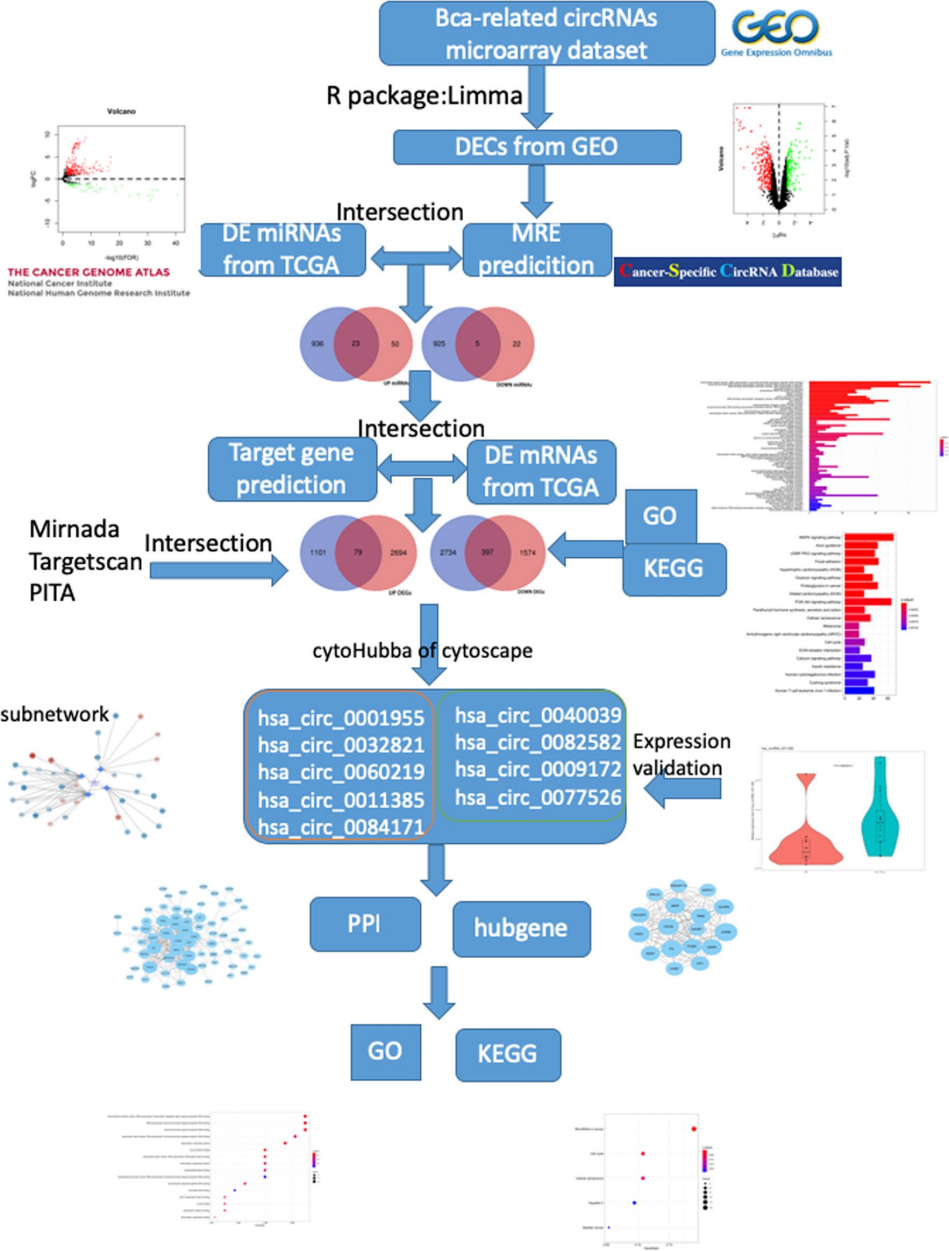
Flowchart of the study. First, we downloaded microarray datasets from GEO to obtain DECs. Next, we intersected miRNAs that they sponge and differentially expressed miRNAs obtained from TCGA. Using similar methods, we obtained overlapping mRNAs and analyzed them for GO and KEGG analyses. Then, we selected nine candidate circRNAs and detected their expression by qRT-PCR. A regulatory network was successfully constructed around hsa_circ_0011385. Subsequently, we detected the expression of target miRNAs and identified hubgenes

In this study, we investigated distinct circRNAs and their potential biological and pathological mechanisms in BCa by using a multi-strategy that combines computational biology and gene chip. A flowchart summarizing our work is presented in Fig. 1. First, we downloaded microarray datasets with information on circRNA expression of BCa from the Gene Expression Omnibus (GEO). Then, we obtained differentially expressed circRNAs (DECs) with R package Limma. Next, we intersected miRNAs that they sponge and differentially expressed miRNAs obtained from The Cancer Genome Atlas (TCGA). Using similar methods, we obtained overlapping messenger RNAs (mRNAs) and used them for Gene Oncology (GO) and Kyoto Encyclopedia of Genes and Genomes (KEGG) analyses. Furthermore, we selected nine candidate circRNAs and detected their expression in BCa tissues and adjacent normal tissues by quantitative real-time polymerase chain reaction (qRT-PCR). Hsa_circ_0011385 was confirmed finally. A circRNA–miRNA–mRNA network was successfully constructed around hsa_circ_0011385. Subsequently, we detected the expression of target miRNAs and identified hubgenes after establishing the protein–protein interaction (PPI). GO and KEGG analyses were performed on the hubgenes. The results provide significant insights into the molecular mechanisms that regulate the tumorigenesis and progression of BCa.

## Methods

### Identification of DECs in BCa from GEO

Microarray dataset GSE92675 (four pairs of BCa and four pairs of adjacent non-cancerous tissues) was retrieved from GEO (http://www.ncbi.nlm.nih.gov/gds/), which is an international public repository for high-throughput microarray and next-generation sequence functional genomic data sets submitted by the research community [13]. The expression profile of circRNAs was determined from GSE92675, and the data were normalized and log10-transformed with the Limma bioconductor package. We selected upregulated circRNAs with the standard of |log10(foldchange) > 3|, adjusted P-value of < 0.01, and downregulated circRNAs with the standard of |log10(foldchange) > 2|, adjusted P-value of < 0.01.

### Differentially expressed miRNAs and mRNAs from TCGA data

We determined the expression levels of miRNAs and mRNAs from TCGA to select differentially expressed miRNAs and mRNAs on the basis of the criteria of |log10(foldchange) > 1|, adjusted P-value of < 0.05.

### Prediction of circRNA–miRNA pairs

CircRNAs function as sponges for miRNAs with MREs. The DECs in BCa that act as sponges for miRNAs were predicted with the cancer-specific circRNA database (CSCD), an online database. Then, RNA sequencing data (composed of 411 BCa samples and 19 normal controls) were downloaded from TCGA [14] to detect differentially expressed miRNAs.

### Prediction of miRNA–mRNA pairs

Three algorithms (miRanda, TargetScan, PITA) were used to depict miRNA target genes. Differentially expressed mRNAs were selected using the same methods for selecting differentially expressed miRNAs.

### GO and KEGG enrichment analyses

ClusterProfiler, an R package that automates the process of biological term classification and enrichment analysis of gene clusters, was used to conduct GO annotation and KEGG pathway analyses [15].

### Tissue samples

Sixteen pairs of BCa tissues and ANTs were obtained from patients who were diagnosed with BCa and underwent radical cystectomy at the First Affiliated Hospital of Nanjing Medical University, China. All tissue samples were frozen in liquid nitrogen until RNA extraction. The experiment was approved by the ethics committee of the First Affiliated Hospital of Nanjing Medical University. Informed consents were signed before the clinical materials were used for research purposes.

### RNA extraction and qRT-PCR

Total RNA was isolated from tissues by using TRIzol reagent (Invitrogen, USA). Then, cDNA was synthesized with HiScript II (Vazyme, China) for qRT-PCR. Primers for qRT-PCR, which was conducted on an AB7300 thermo-recycler (Applied Biosystems, USA) or LightCycler 480 (Roche, USA), were provided by TSINGKE Biological Technology. U6 and beta-actin were used as the internal reference. Expression levels of circRNAs were calculated using the 2^−ΔΔCT^ method.

### Subnetwork construction of the circRNA and PPI network

The cytoHubba plugin from Cytoscape 3.7.1 was used to select the functional circRNA–miRNA–mRNA network according to the degree value.

## Results

### Identification of DECs in BCa

**Fig. 2:**
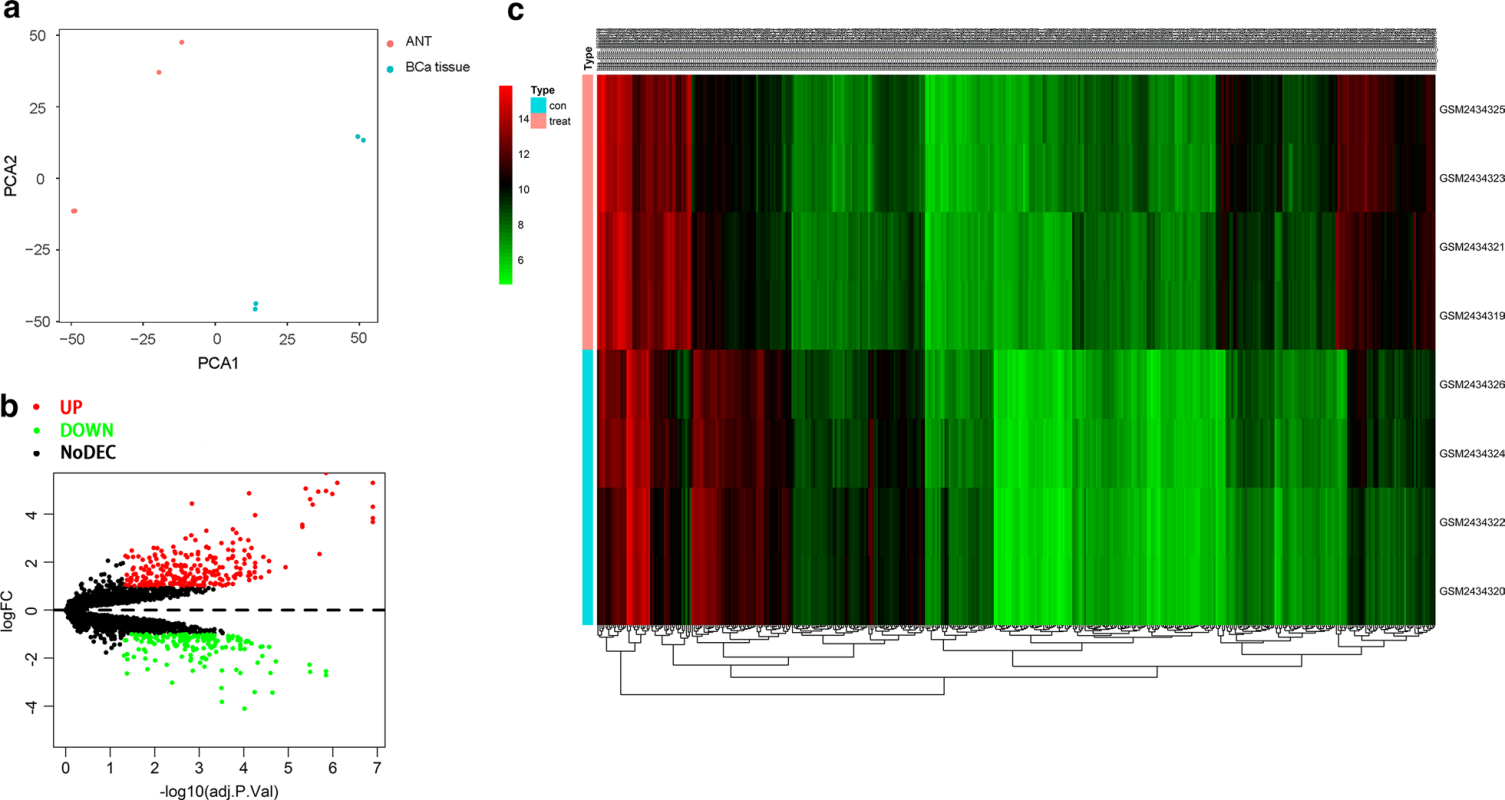
**a** The PCA plot showed the PC1 and PC2 coefficients of GSE92675. **b** Volcano plot for DECs in BCa on the basis of GSE92675. A total of 428 DECs were identified, which consisted of 261 upregulated and 167 downregulated circRNAs. We ranked all DECs using a robust method and an adjusted P-value of < 0.05. Twenty-one upregulated and 28 downregulated circRNAs were in the top rankings. **c** Heatmap for DECs in BCa on the basis of GSE92675

A microarray dataset (GSE92675) from the GPL19978 platform was utilized in this study. Table 1 shows the fundamental information of the gene chip. To select DECs, R package Limma was adopted. The PCA plot showed the PC1 and PC2 coefficients of GSE92675 (Fig. 2a). As a result, 428 DECs were identified, which consisted of 261 upregulated and 167 downregulated circRNAs (Fig. 2b). We ranked all DECs using a robust method and an adjusted P-value of < 0.05. Twenty-one upregulated and 28 downregulated circRNAs were in the top rankings. The heat map for circRNAs in GSE92675 is shown in Fig. 2c.

**Table 1:** Basic information of the microarray dataset from GEO

Data source	Platform	First author	Year	Region	Sample size (T/N)
GSE92675	GPL19978	Chen J	2016	CHINA	4/4

*GEO* Gene Expression Omnibus, *T* tumor, *N* normal

### Construction of the circRNA–miRNA–mRNA network in BCa

**Fig. 3:**
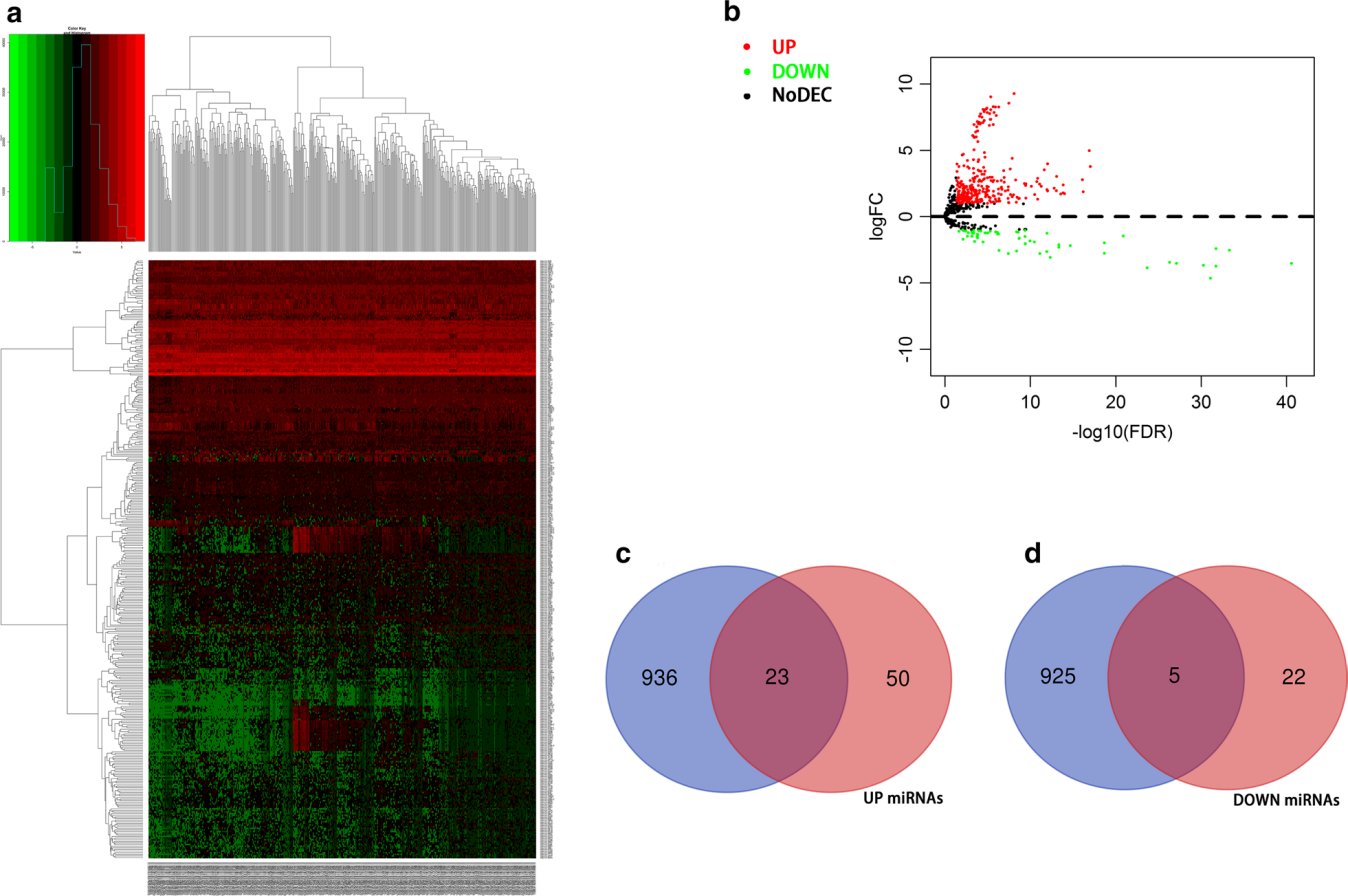
**a**, **b** Differentially expressed miRNAs were obtained from TCGA. **c**, **d** We confirmed 28 target miRNAs by intersecting target miRNAs from CSCD and differentially expressed miRNAs obtained from TCGA

**Fig. 4:**
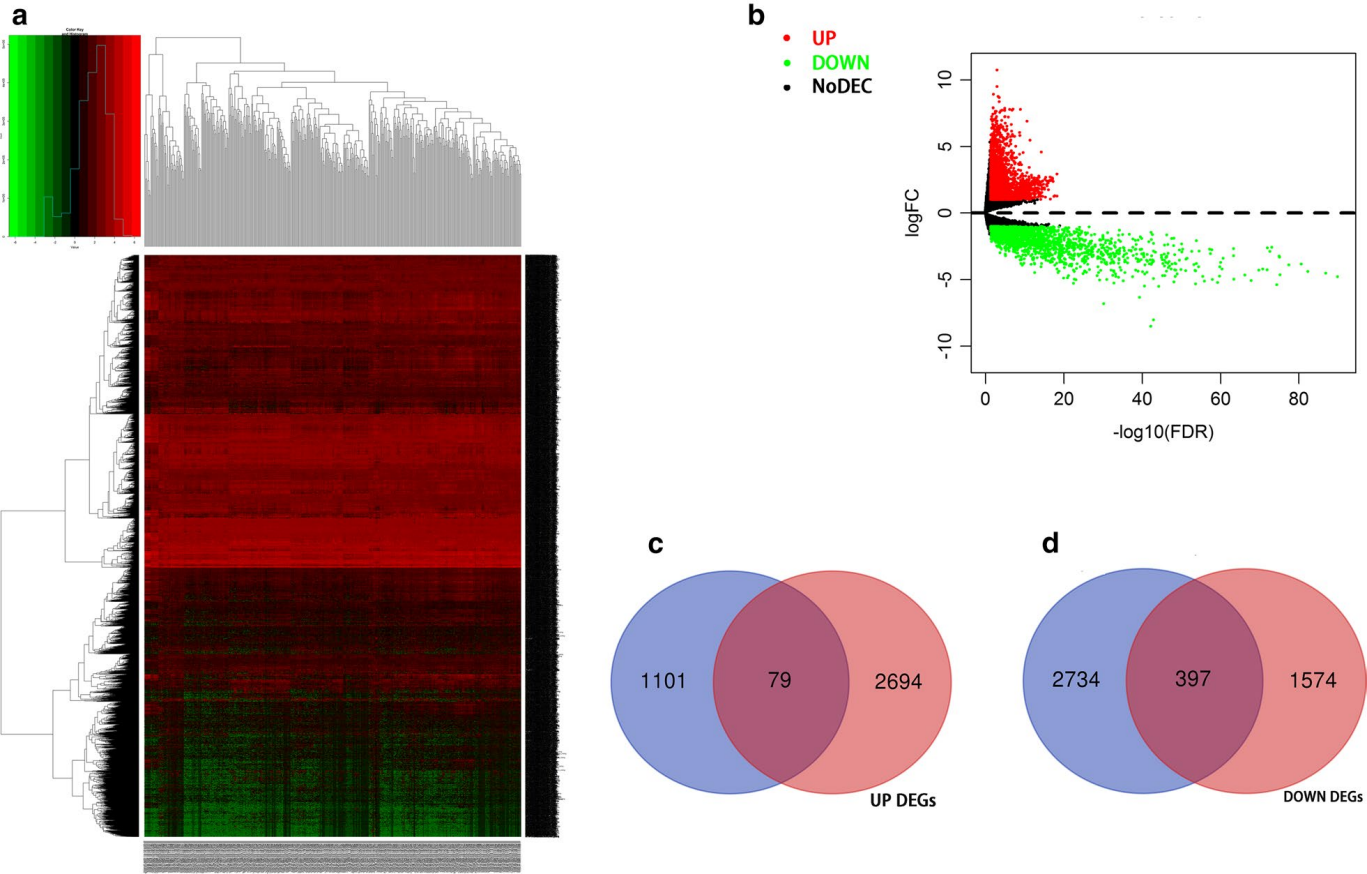
**a**, **b** Differentially expressed mRNAs were obtained from TCGA. **c**, **d** By intersecting target mRNAs and differentially expressed mRNAs, 476 target mRNAs were selected, of which 79 were upregulated and 397 were downregulated

**Fig. 5:**
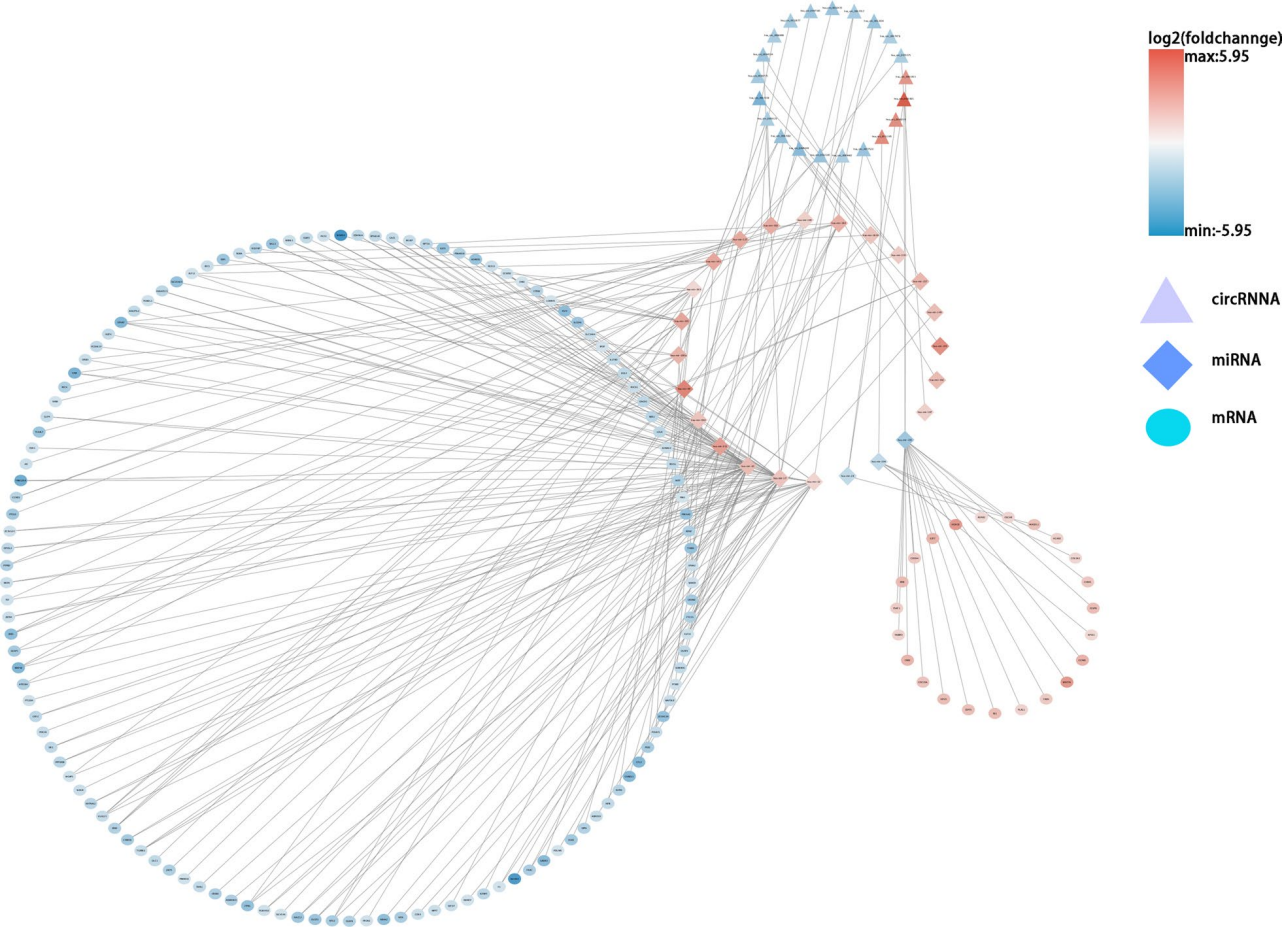
CircRNA–miRNA–mRNA regulatory network for DECs

**Fig. 6:**
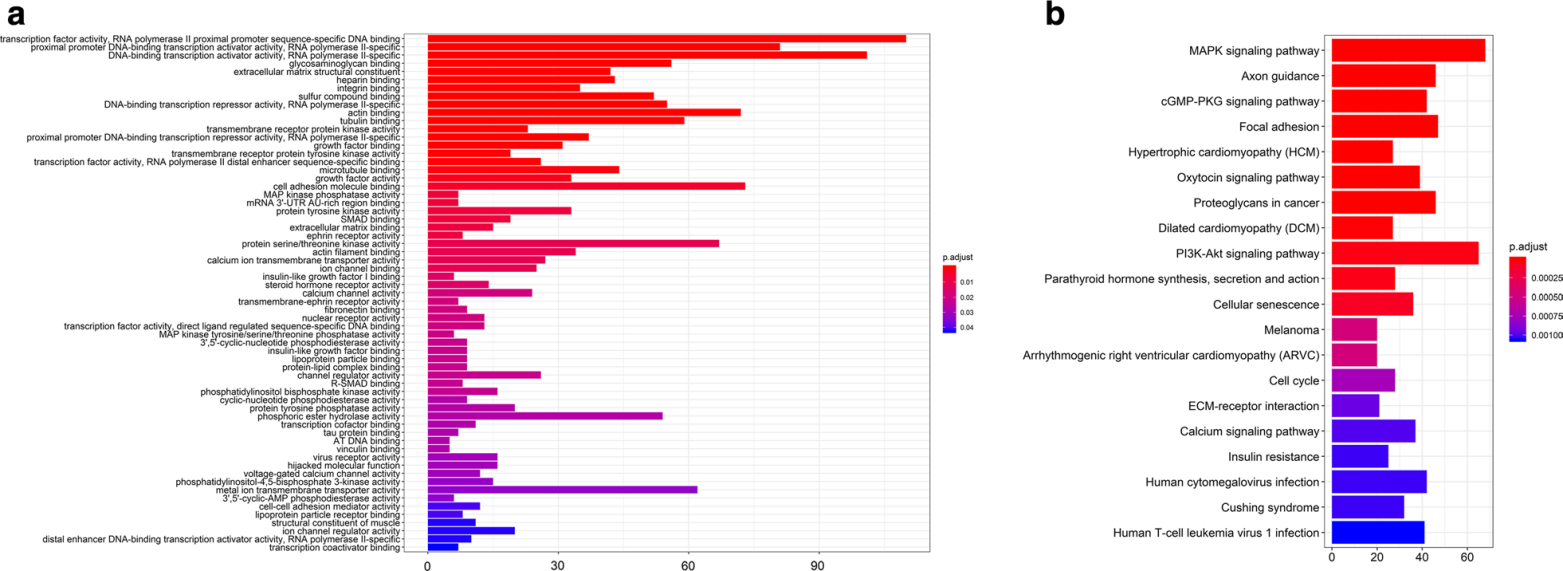
**a**, **b** GO and KEGG analyses were conducted to detect the potential biological functions of target mRNAs. The results showed that the MAPK and PI3K–AKT signaling pathways were statistically significant

On the basis of the above results, we conducted circRNA–miRNA pair prediction using CSCD. A total of 1861 target miRNAs were obtained. By intersecting target miRNAs and differentially expressed miRNAs obtained from TCGA, we confirmed 28 target miRNAs finally (Fig. 3). Using similar methods, 476 target mRNAs were selected, of which 79 were upregulated and 397 were downregulated (Fig. 4). Finally, the circRNA–miRNA–mRNA network containing circRNA–miRNA pairs and miRNA–mRNA pairs was generated (Fig. 5). Next, we conducted GO and KEGG analyses to detect the potential biological functions. The results are shown in Fig. 6. The MAPK and PI3K–AKT signaling pathways were statistically significant.

### Subnetwork analyses of circRNA

**Fig. 7:**
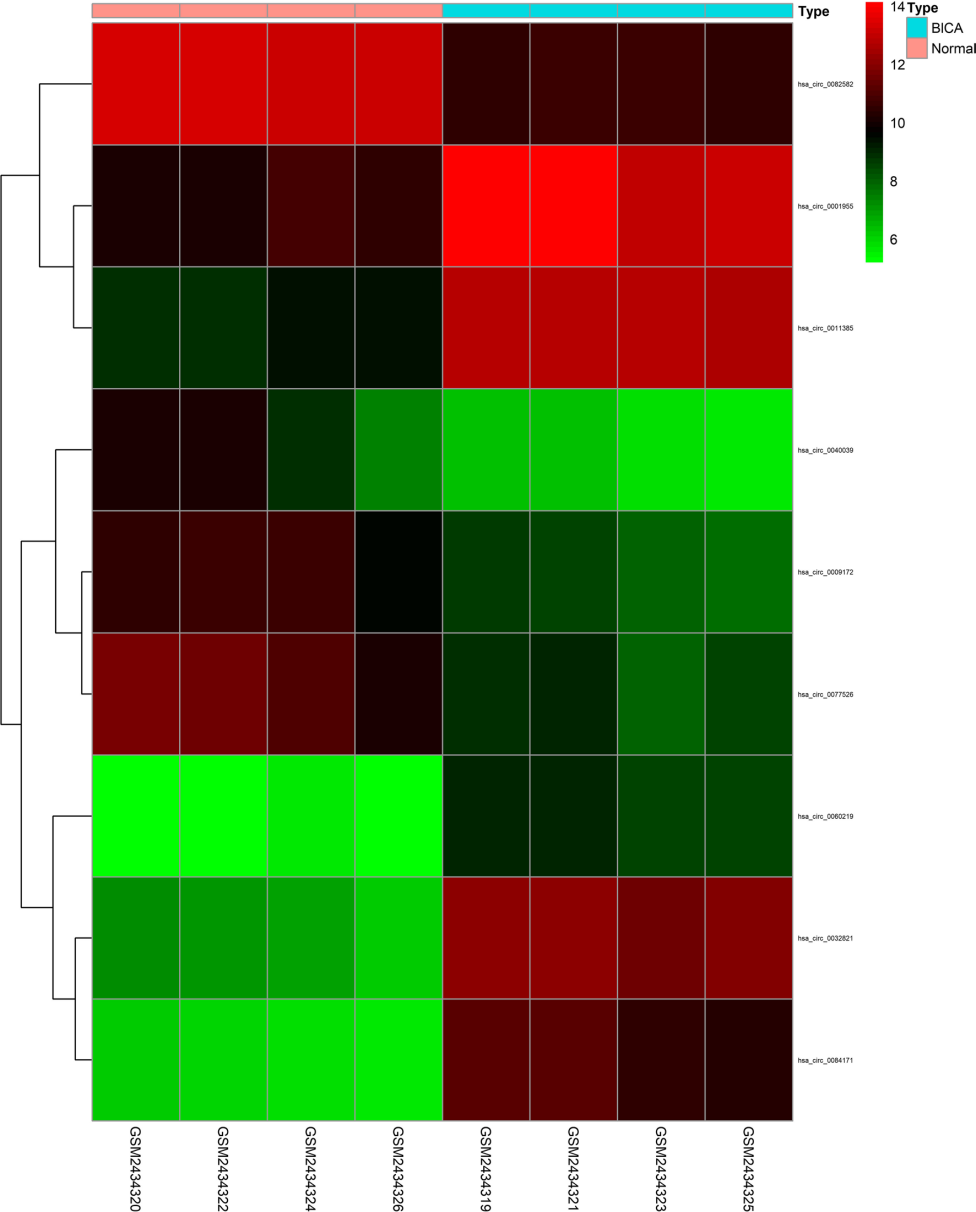
Heatmap for nine circRNAs (hsa_circ_0001955, hsa_circ_0032821, hsa_circ_0060219, hsa_circ_0011385, hsa_circ_0084171, hsa_circ_0040039, hsa_circ_0082582, hsa_circ_0009172, hsa_circ_0077526) in GSE92675

**Fig. 8:**
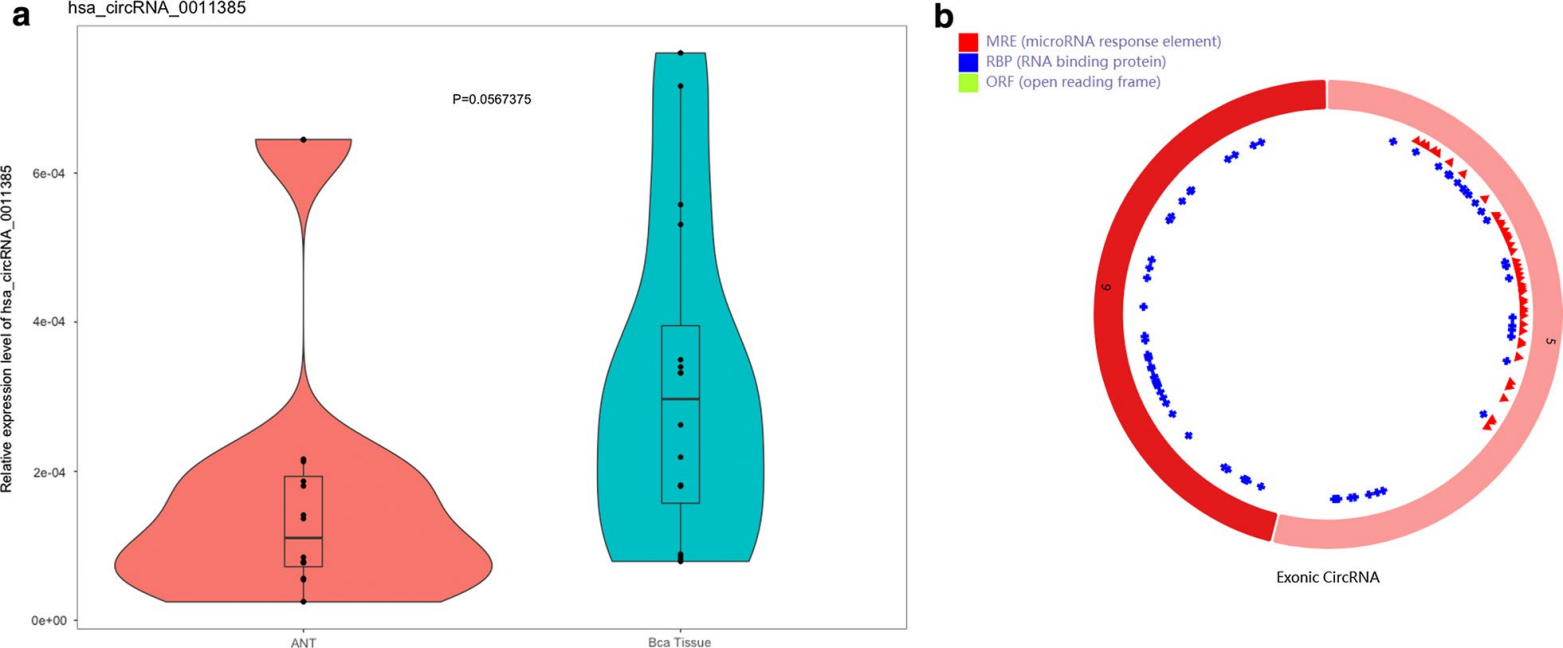
**a** Violin plot for the expression of hsa_circ_0011385. **b** Structural patterns of hsa_circ_0011385 from CSCD

**Fig. 9:**
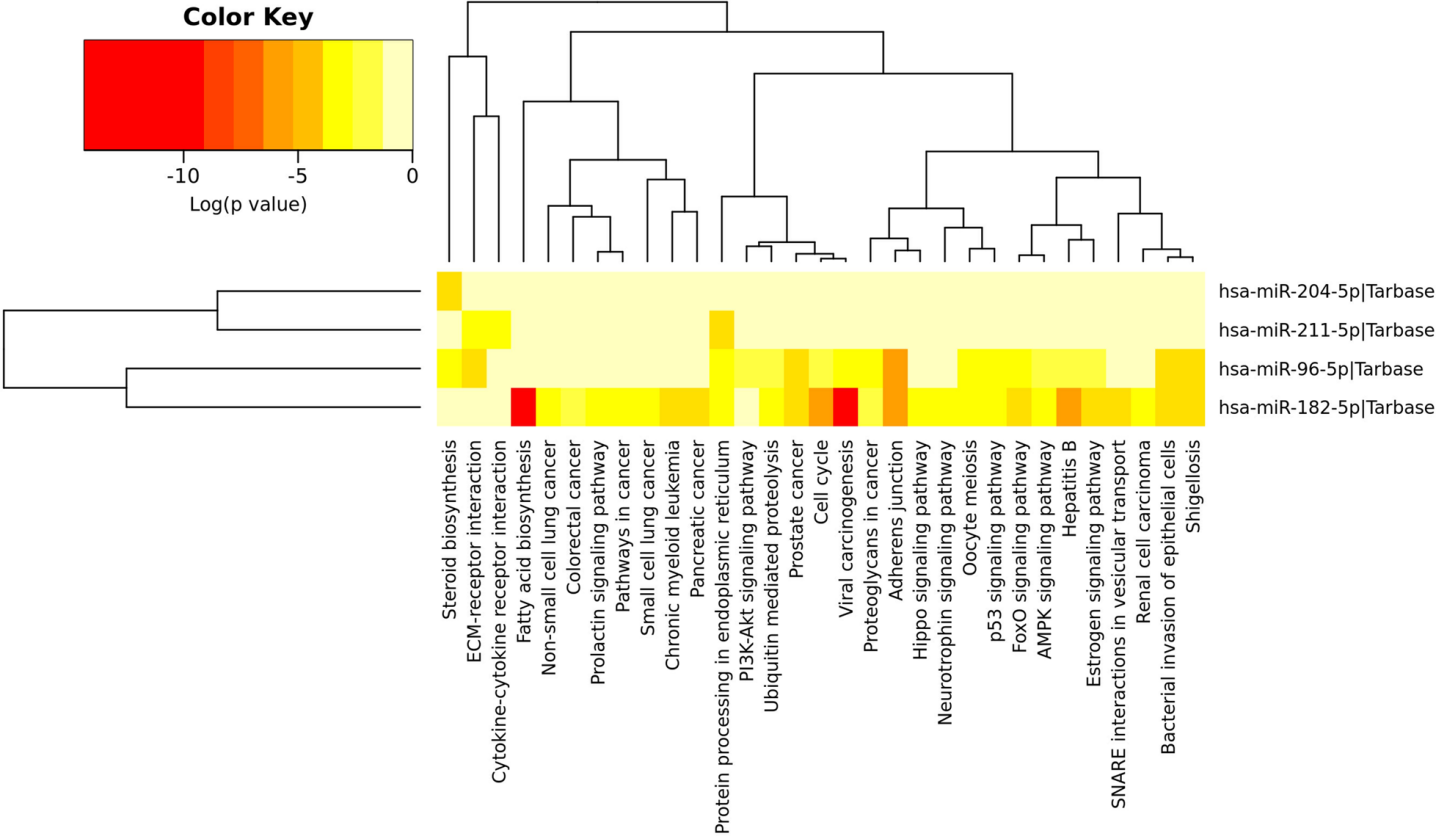
Heatmap for the significant signaling pathways mediated by the four miRNAs (mir-211-5p, mir-204-5p, mir-182-5p, mir-96-5p) according to DIANA-miRPath

**Fig. 10:**
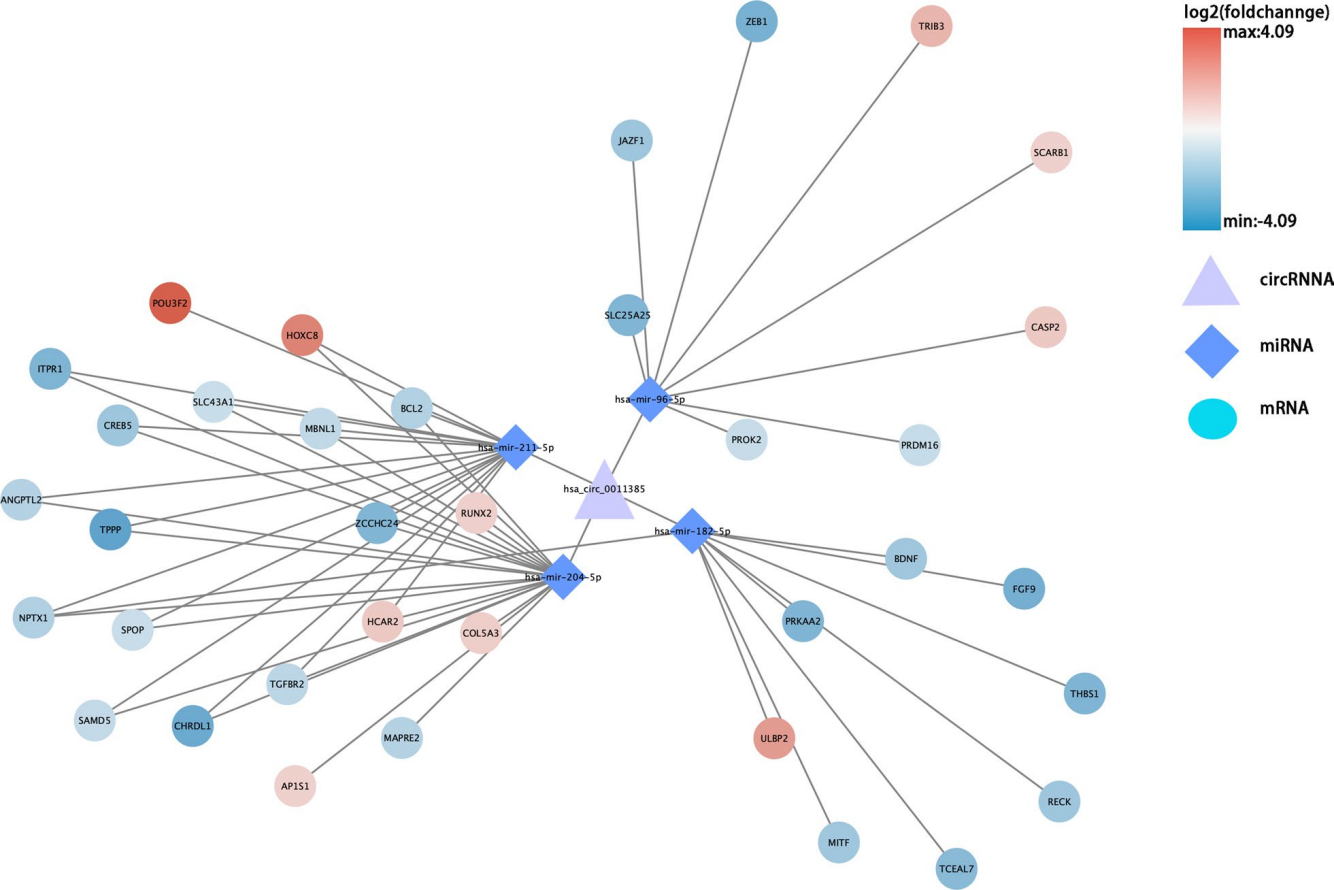
CircRNA–miRNA–mRNA regulatory network for hsa_circ_0011385

**Fig. 11:**
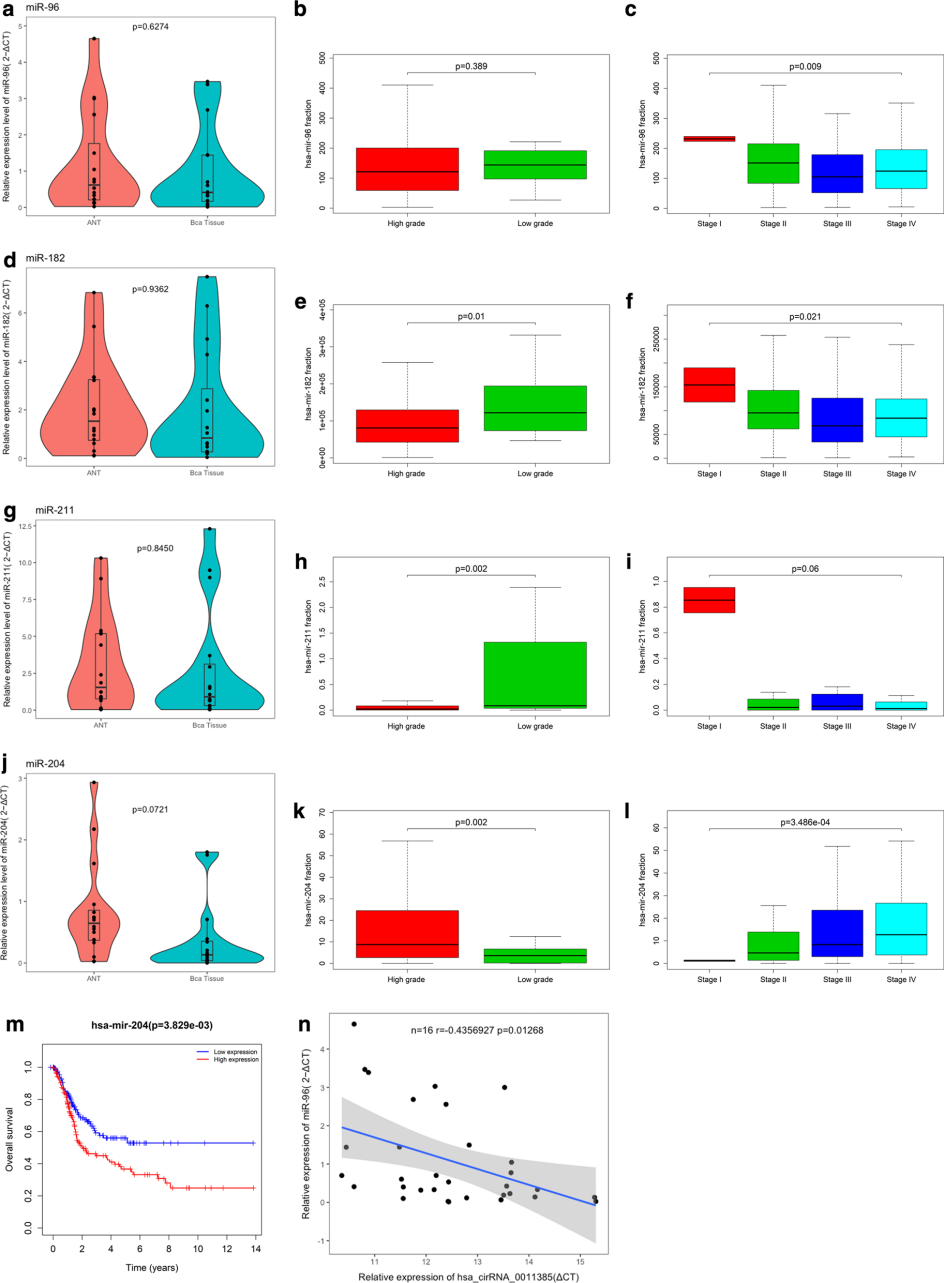
**a**, **d**, **g**, **i** Violin plots for the expression of mir-96-5p, mir-182-5p, mir-211-5p, and mir-204-5p in 16 BCa patients. **b**, **e**, **h**, **k** Differentially expressed miRNAs between high-grade and low-grade BCa except mir-96-5p. **c**, **f**, **i**, **l** Mir-96-5p, mir-182-5p, mir-211-5p, and mir-204-5p showed significant difference in different stages of BCa. **m** High expression of mir-204-5p was correlated with poor prognosis in BCa. **n** Correlation analysis revealed a moderate negative correlation between the expression of hsa_circ_0011385 and miR-204 (r = − 0.43, P = 0.01268)

CircRNAs act as hub nodes in biological networks. Five downregulated circRNAs (hsa_circ_0001955, hsa_circ_0032821, hsa_circ_0060219, hsa_circ_0011385, hsa_circ_0084171) and four upregulated circRNAs (hsa_circ_0040039, hsa_circ_0082582, hsa_circ_0009172, hsa_circ_0077526) were identified on the basis of the degree value calculated by the cytoHubba plugin of Cytoscape. The heatmap for nine DECs in GSE92675 is shown in Fig. 7, and their basic features are displayed in Table 2. Sequences of specific primers for each circRNA were designed using Primer Premier 5. The expression levels of nine circRNAs in 16 pairs of BCa tissues and adjacent non-cancerous tissues were quantified by qRT-PCR. Among the qRT-PCR products, five circRNAs were unable to be verified by agarose gel electrophoresis. Two circRNAs could not be detected due to their relatively low expression. The expression of hsa_circ_0060219 showed no significant difference between BCa tissues and adjacent non-cancerous tissues. Hsa_circ_0011385 showed upregulated tendency in BCa tissues corroborated by qRT-PCR (Fig. 8a). On the basis of the above results, hsa_circ_0011385 was selected as the target for future research. The basic structural molds of hsa_circ_0011385 are displayed in Fig. 8b. Clinicopathological features in 16 BCa patients are displayed in Table 3. Four downregulated miRNAs (mir-211-5p, mir-204-5p, mir-182-5p, mir-96-5p) were confirmed. All four miRNAs were involved in cancer-related pathways as shown in Fig. 9. After obtaining the overlapping genes from target mRNAs in BCa and differentially expressed mRNAs from TCGA, a circRNA–miRNA–mRNA network was constructed around hsa_circ_0011385 (Fig. 10). Afterward, the expression of four miRNAs was validated by qRT-PCR as well. Mir-96-5p, mir-182-5p, and mir-211-5p showed no significant difference (Fig. 11a, d, g), whereas mir-204-5p showed downregulated tendency (Fig. 11j). The primer information is shown in Table 4. Correlation analysis revealed a moderate negative correlation between the expression of hsa_circ_0011385 and miR-204 (r = − 0.43, P = 0.01268) (Fig. 11n). Furthermore, according to the data from TCGA, we found that those miRNAs were differently expressed between high-grade and low-grade BCa except mir-96-5p (Fig. 11b, e, h, k). However, mir-96-5p, mir-182-5p, mir-211-5p, and mir-204-5p showed significant difference in different stages of BCa (Fig. 11c, f, i, l). High expression of mir-204-5p was correlated with poor prognosis in BCa (Fig. 11m).

**Table 2:** Features of 9 selected circRNAs

circRNA name	Gene symbol	Position	Strand	Regulation
hsa_circ_0001955	CSNK1G1	chr15:64495280–64508912	−	Up
hsa_circ_0032821	CEP128	chr14:81209418–81227957	−	Up
hsa_circ_0060219	KIAA0889	chr20:35457456–35467844	−	Up
hsa_circ_0011385	EIF3I	chr1:32691771–32692131	+	Up
hsa_circ_0084171	FNTA	chr8:42914234–42932507	+	Up
hsa_circ_0040039	SNTB2	chr16:69279504–69318147	+	Down
hsa_circ_0082582	TRIM24	chr7:138203933–138255748	+	Down
hsa_circ_0009172	DNA2	chr10:70218860–70229920	−	Down
hsa_circ_0077526	BVES	chr6:105563560–105564743	−	Down

**Table 3:** Clinicopathological features in 16 BCa patients

Characteristics	Case	hsa_circ_0011385 expression		P value
		Low	High	
All cases	16	8	8	
Age (years)				
< 65	10	6	4	0.6084
≥ 65	6	2	4	
Gender				0.8287
Male	7	4	3	
Female	9	4	5	
Smoke				0.1626
Yes	13	7	6	
No	3	1	2	
Grade				0.8022
Low	7	3	4	
High	9	5	4	
Tumor size(cm)				0.0406*
< 3	9	2	7	
≥ 3	7	6	1	

*P < 0.05

**Table 4:** Primers sequences for qRT-PCR

Gene ID	Primer sequences	
	Forward	Reverse
Beta-actin	AGCGAGCATCCCCCAAAGTT	GGGCACGAAGGCTCATCATT
hsa_circ_0011385	TCATTGTTGTCAATCTGGCTC	CAGTGCTGTTTGGGGACC
U6	AAAGCAAATCATCGGACGACC	GTACAACACATTGTTTCCTCGGA
mir-211-5p	CGCGTCCGCTTCCTACTGTT	AGTGCAGGGTCCGAGGTATT
mir-204-5p	GCGCGTCCGTATCCTACTGTT	AGTGCAGGGTCCGAGGTATT
mir-182-5p	CGCGTCACACTCAAGATGGTAA	AGTGCAGGGTCCGAGGTATT
mir-96-5p	CGCGTCGTTTTTACACGATCA	AGTGCAGGGTCCGAGGTATT

### Identification of hubgenes from the PPI network using the Molecular Complex Detection (MCODE) algorithm

**Fig. 12:**
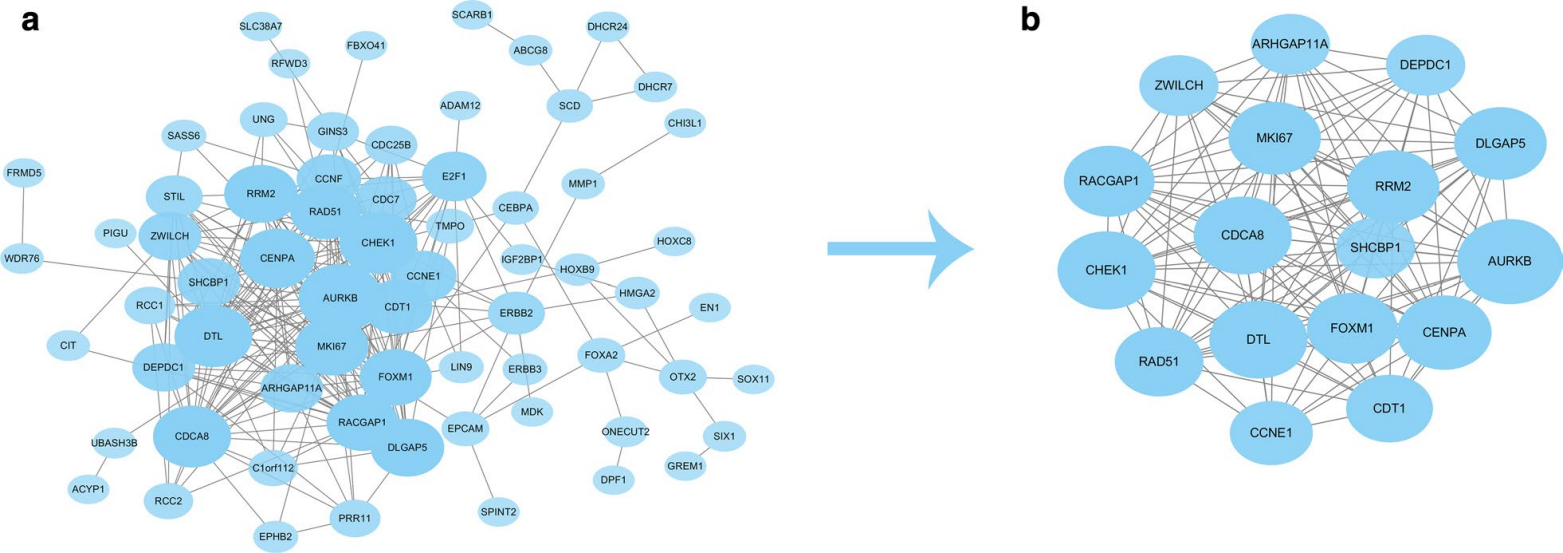
**a** PPI network of target genes. **b** PPI network of the 18 hubgenes extracted from a

**Fig. 13:**
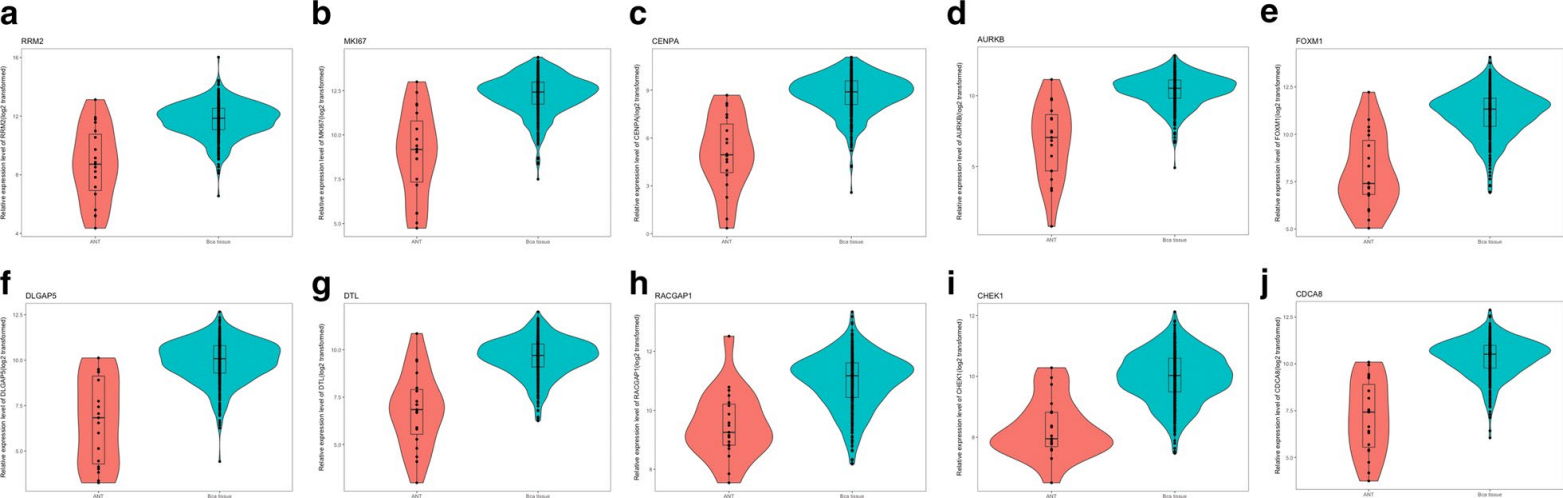
Violin plots for the expression of the top 18 hubgenes on the basis of TCGA. **a** RRM2, **b** MKI67, **c** CENPA, **d** AURKB, **e** FOXM1, **f** DLGAP5, **g** DTL, **h** RACGAP1, **i** CHEK1, **j** CDCA8

**Fig. 14:**
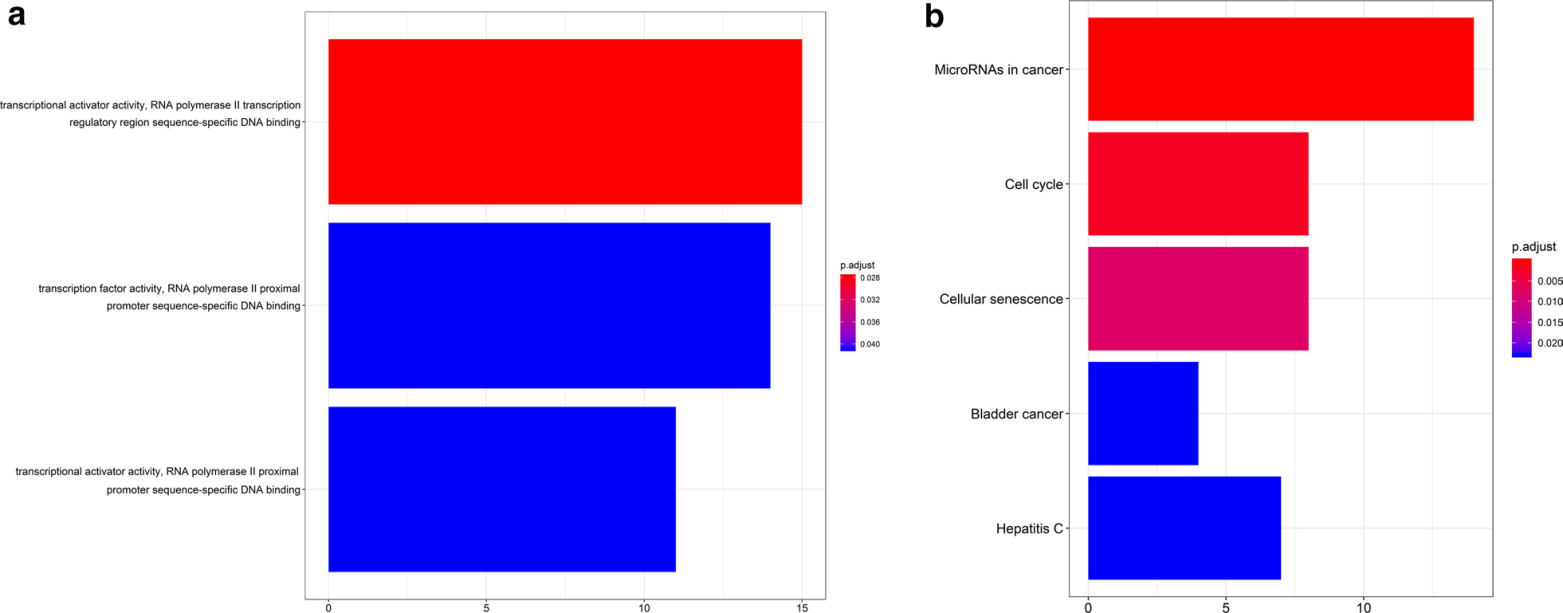
**a**, **b** GO and KEGG analyses were conducted to detect the potential biological functions of hubgenes

After obtaining the target genes of hsa_circ_0011385, we created a PPI network composed of 67 nodes and 274 edges (Fig. 12a). Following the identification of the vital functions of hubgenes in the network, 18 hubgenes (ARHGAP11A, DEPDC1, DLGAP5, AURKB, CENPA, CDT1, CCNE1, RAD51, CHEK1, RACGAP1, ZWILCH, MKI67, CDCA8, DTL, FOXM1, SHCBP1, RRM2, MKI67) were identified in BCa using the MCODE algorithm, k-score = 2. The expression levels of the top 10 hubgenes (RRM2, MKI67, CENPA, AURKB, FOXM1, DLGAP5, DTL, RACGAP1, CHEK1, CDCA8) are shown in Fig. 13. A circRNA–miRNA–hubgene network is displayed in Fig. 12b. GO and KEGG analyses showed that hubgenes were involved in cell cycle activities and could be regulated by miRNAs and play an eventful role in BCa pathogenesis (Fig. 14).

## Discussion

CircRNA, a novel class of ceRNA, is resistant to nuclease due to its covalently closed loop structure without 5′ to 3′ polarity or a polyadenylated tail [16]. The advent of high-throughput sequencing technology and development of bioinformatics have had a significant impact on the study of circRNAs. However, the role of circRNAs in cancer progression has not been explained clearly. Numerous studies have shown that the expression profiles of circRNAs are abnormal in many types of cancers. Together with miRNAs and their target genes, the circRNA–miRNA–mRNA axis can function as a broad network of gene expression regulators, and it is able to serve as diagnostic and prognostic biomarkers.

An increasing number of circRNAs, such as cTFRC, circELP3, and circMTO1, have been reported to be closely related to BCa progression [17–19]. For example, Li et al. [17] found that circMTO1 is significantly decreased in BCa tissues compared with adjacent tissues and that the low expression level of circMTO1 is positively linked with metastasis and poor survival in BCa patients. They also revealed that circMTO1 competes for miR-221 and that the ectopic expression of circMTO1 negatively regulates the E-cadherin/N-cadherin pathway to inhibit BCa cells’ epithelial–mesenchymal transition (EMT) by sponging miR-221. Similarly, Su et al. [18] found that cTFRC downregulation inhibits cell invasion and proliferation, reduces EMT, and facilitates tumor growth in vivo by functioning as a ceRNA through harboring miR-107 to abolish the suppressive effect on the target gene TFRC. However, several circRNAs remain to be detected.

In this study, we systematically analyzed the regulatory network comprising circRNAs, miRNAs, and mRNAs in BCa. Then, we downloaded the gene chip, GSE92675, from the GEO database to identify DECs in BCa with a robust method and an adjusted P-value of < 0.05. Twenty-one upregulated and 28 downregulated circRNAs were selected for further research.

Although the mechanism of circRNAs in regulating malignant biological behavior needs to be elucidated, many circRNAs harbor plentiful MREs serving as ceRNAs, demonstrating that they can regulate gene expression by sponging corresponding miRNAs [20]. We predicted potential circRNA–miRNA pairs via CSCD to ascertain their function as ceRNAs. CSCD predicts miRNAs within 50 bp upstream or downstream of the circRNA junction point [21]. Finally, we confirmed 28 target miRNAs by intersecting the predicted miRNAs and differentially expressed miRNAs that were obtained from TCGA. Similarly, we intersected potential target genes with differentially expressed mRNAs to construct a circRNA–miRNA–mRNA regulatory network preliminarily. GO and KEGG analyses showed that the network was involved in many critical tumor-associated biological behaviors and metabolic pathways, such as the MAPK and PI3K–AKT signaling pathways, which are widely reported in regulating bladder carcinogenesis [22, 23].

According to the degree value calculated by the cytoHubba plugin of Cytoscape, nine circRNAs (hsa_circ_0001955, hsa_circ_0032821, hsa_circ_0060219, hsa_circ_0011385, hsa_circ_0084171, hsa_circ_0040039, hsa_circ_0082582, hsa_circ_0009172, hsa_circ_0077526) were selected as the key circRNAs in the network. After being validated by qRT-PCR in 16 pairs of BCa tissues and adjacent non-cancerous tissues, hsa_circ_0011385 was selected as the key circRNA for further analyses. Next, we confirmed four downregulated miRNAs (mir-211-5p, mir-204-5p, mir-182-5p, mir-96-5p). Notably, mir-182-5p has been reported to be associated with BCa pathogenesis [24, 25]. Xie et al. [24] found that BCRC-3 can directly interact with miR-182-5p as a miRNA sponge to promote the miR-182-5p-targeted 3′UTR activity of p27. They suggested that miR-182-5p plays a tumor promoter role by regulating cell cycle progression in BCa cells. Hirata et al. [25] supposed that miR-182-5p acts as an oncogene by knocking down RECK and Smad4, promoting BCa development. We found that mirNA-204 was downregulated in BCa and correlated with the prognosis of patients. After we obtained the overlapping mRNAs between target mRNAs and differentially expressed mRNAs in BCa, we constructed a circRNA–miRNA–mRNA network around hsa_circ_0011385. Hsa_circ_0011385 could regulate gene expression by functioning as a miRNA sponge, indicating its potential regulatory mechanism. We created a PPI network screening 18 hubgenes to clarify the regulatory mechanism of the ceRNA network. GO and KEGG analyses showed that hubgenes could be regulated by miRNAs and play an eventful role in BCa pathogenesis. In addition, they were involved in cell cycle activities, which was consistent with the results of previous studies. We proposed a novel circRNA–miRNA–mRNA network related to BCa pathogenesis. This network might be a new molecular biomarker and could be used to develop potential treatment strategies for BCa.

However, because the results were based on computational biology and qRT-PCR, in vitro or in vivo biological and molecular experiments need to be conducted to verify our hypothesis.

## Conclusions

We proposed a novel circRNA–miRNA–mRNA regulatory network after analyzing DECs, differentially expressed miRNAs, and differentially expressed mRNAs by qRT-PCR and computational biology. We found that hsa_circ_0011385 may function as a ceRNA, which plays a critical role in carcinogenesis-related pathways, providing new avenues for mechanistic investigation and offering a potential biomarker for BCa. Further studies are needed to detect the role of regulatory modules in the carcinogenesis of BCa.

## Data Availability

Authors can provide all of datasets analyzed during the study on reasonable request.

## Abbreviations

BCa: bladder cancer
ceRNA: competing endogenous RNA
circRNA: circular RNA
CSCD: cancer-specific circRNA database
DEC: differently expressed circRNAs
DE mRNA: differently expressed mRNA
DE miRNA: differently expressed miRNA
FDR: adjusted *P* value
GEO: Gene Expression Omnibus
GO: Gene Oncology
KEGG: Kyoto Encyclopedia of Genes and Genomes
MIBC: muscle-invasive bladder cancer
MRE: miRNA response elements
MCODE: Molecular Complex Detection
NMIBC: non-muscle-invasive bladder cancer
PPI: protein-protein interaction
qRT-PCR: quantitative real-time polymerase chain reaction
TCGA: The Cancer Genome Atlas
